# The treatment of bullous pemphigoid with topical roflumilast 0.3% cream case study

**DOI:** 10.1016/j.jdcr.2026.02.005

**Published:** 2026-02-10

**Authors:** Erik Domingues

**Affiliations:** a Modern Dermatology of Massachusetts, Fall River, Massachusetts; b Department of Dermatology, Assistant Professor, University of Massachusetts Chan Medical School, Worcester Massachusetts

**Keywords:** autoimmune blistering disorder, bullae, bullous pemphigoid (BP), erosions, PDE-4 inhibitor, phosphodiesterase-4 (PDE-4) inhibitor, roflumilast cream 0.3%, topical

## Introduction

Bullous pemphigoid (BP), the most common autoimmune blistering disorder, is characterized by autoantibodies targeting the dermal-epidermal junction (DEJ), leading to pruritic, urticarial plaques and tense bullae.^1^, ^2^, ^3^ BP significantly impairs quality of life through pruritus, painful lesions, and treatment burdens including potential adverse events from common therapies.^1^^,^^3^ While topical and oral corticosteroids remain the first line of treatment for BP, their potential adverse effects have encouraged the use of steroid-sparing immunosuppressants.^1^^,^^3^ In June 2025, Dupilumab was approved by the US Food and Drug Administration (FDA) for the treatment of BP, making it the first targeted therapy for this disease.^4^ Non-steroidal, and potent topical phosphodiesterase-4 (PDE4) inhibitor, roflumilast, has been FDA approved for several inflammatory dermatoses, establishing its efficacy and safety in plaque psoriasis, atopic dermatitis, and seborrheic dermatitis.^5^

This case study describes a 93-year-old male who presented with a pruritic, erythematous eruption for 2 months unresponsive to topical corticosteroids and tacrolimus 0.1% ointment. He demonstrated marked clinical improvement following treatment with roflumilast 0.3% cream, and noteworthy resolution of severe pruritus within 24 hours of first application of roflumilast cream.

## Case report

**Fig 1:**
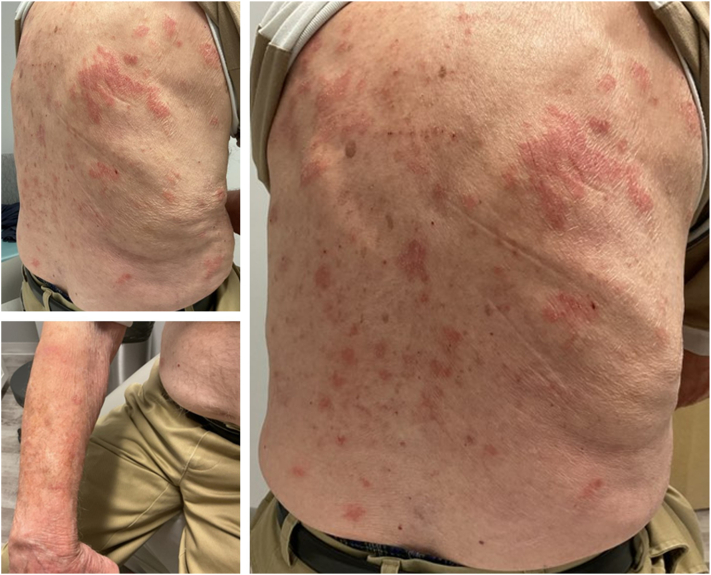
Baseline photos prior to treatment with roflumilast 0.3% cream.

**Fig 2:**
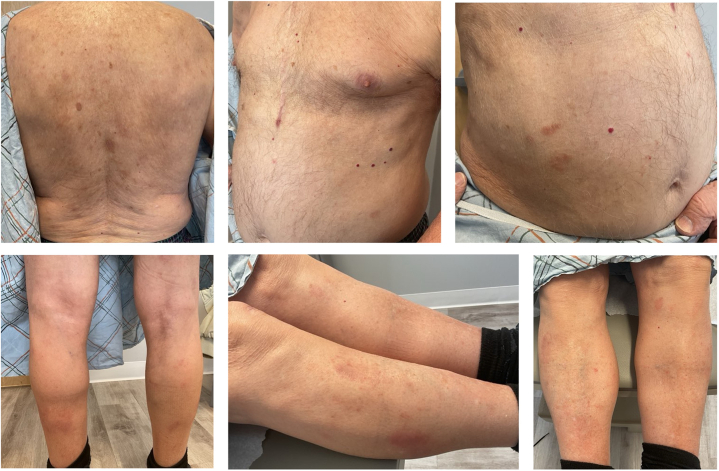
Follow up visit 6 weeks after initiation of roflumilast 0.3% cream.

We report the case of a 93-year-old male who presented with a 2-month history of a pruritic, erythematous eruption unresponsive to hydrocortisone 1% cream and triamcinolone 0.1% cream. Physical examination revealed erythematous, eczematous plaques involving 10% body surface area (BSA), predominantly on the trunk and extremities (Fig 1). He reported significant pruritus (7/10). Treatment options were discussed, and he was prescribed tacrolimus ointment 0.1% twice daily (BID) to affected areas. He reported no improvement with tacrolimus after 6 weeks. Two punch biopsies were performed on the left posterior upper arm, including 1 for direct immunofluorescence (DIF). Lesional histology showed spongiotic dermatitis with eosinophils. DIF revealed 2+ linear depositions of C3, IgG, and IgG4 at the basement membrane zone consistent with pemphigoid. Recommended treatment options such as doxycycline and clobetasol ointment were declined by the patient. He continued tacrolimus ointment BID while approval for Dupilumab was pending but was eventually unable to initiate due to access issues. On follow-up, his dermatosis persisted and pruritus worsened to 10/10. He was instructed to discontinue tacrolimus and given his age, prednisone and other immunosuppressives were avoided to minimize adverse effects. He was given roflumilast 0.3% cream to be applied once daily. Six weeks later, he stated pruritus had resolved within 24 hours of first application. Further examination revealed residual post-inflammatory hyperpigmentation on the torso and extremities with a few resolving pink eczematous papules and plaques on the abdomen and lower legs (Fig 2). Pruritus and affected BSA decreased to 0/10 and 1%, respectively, without adverse reactions. The patient was instructed to use roflumilast cream as needed for any pruritic or active papules and plaques. At a recent 6-month follow-up, he has remained clear without recurrence of BP.

## Discussion

This case report describes a 93-year-old male with bullous pemphigoid treated with roflumilast 0.3% cream as monotherapy. Pruritus nearly resolved within 24 hours of first application, and his dermatosis showed near-complete resolution after failing previous topical corticosteroids and calcineurin inhibitor therapies. In this case, once the biopsy confirmed BP and he declined oral antibiotics and additional topical steroids, an alternative topical therapy was considered. Roflumilast cream 0.3% was initiated as a targeted, steroid-free anti-inflammatory treatment with known anti-pruritic effects. This case underscores the potential role of roflumilast cream as a valuable therapeutic alternative for patients with BP, especially those with contraindications to systemic therapies.

In this case, the diagnosis of BP was made based on the gold-standard DIF result and classic clinical presentation, which was deemed sufficient for clinical decision-making. Of note, however, the absence of serological confirmation by indirect immunofluorescence or antigen-specific ELISA represents a diagnostic limitation, as it is the most specific diagnostic test for BP and helps to differentiate from other subepidermal blistering disorders and less common pemphigoid variants.

The selection of roflumilast was based on its potent anti-inflammatory properties as a PDE4 inhibitor and its predictable, favorable safety profile. Its mechanism of action has attracted increased interest in PDE4 as a potential target in inflammatory diseases, and I believe this case study suggests it may be particularly relevant in the context of autoimmune blistering diseases such as BP. PDE4 inhibition elevates intracellular cyclic AMP, leading to the downregulation of pro-inflammatory mediators and cytokines, and a decrease in neutrophil activation. There are 2 published studies in mouse models of epidermolysis bullosa acquisita (EBA), a similar autoimmune blistering disease that often mimics BP. One experimental in vivo study demonstrated that PDE4 inhibition impaired both disease manifestation and progression of EBA.^6^ This supports the mechanistic relevance of the PDE4 drug class in subepidermal blistering diseases. Another study indicated that PDE4 is involved in promoting inflammation and blistering.^7^ In vivo, roflumilast was initiated after the onset of skin inflammation in immunized mice, and not only halted disease progression, but also demonstrated efficacy equivalent to high-dose corticosteroids.^7^

Clinical trials in BP are lacking, and while this is a single case, the observed clinical response coincided with the initiation of roflumilast cream. Although spontaneous improvement remains a possibility, the collective evidence provides a rationale for the response observed in this patient, and suggests that topical PDE4 inhibition may represent a promising and targeted therapeutic avenue worthy of further investigation in pemphigoid diseases, including BP. This case report highlights roflumilast 0.3% cream as a potential, well tolerated, steroid-free option for BP, but robust clinical studies are necessary to confirm its efficacy.

## Conflicts of interest

ED is a speaker, advisor, and/or consultant for Abbvie, Apogee, Arcutis, BMS, Castle Biosciences, Galderma, Incyte, Johnson and Johnson, Kowa, Leo Pharma, Pelthos, Pfizer, UCB, and Veradermics.
